# Profiling of patients with treatment-resistant schizophrenia: a retrospective analysis

**DOI:** 10.1192/j.eurpsy.2025.2254

**Published:** 2025-08-26

**Authors:** S. Ben Aissa, K. Razki, C. Najar, A. Larnaout, W. Melki

**Affiliations:** 1 Psychiatry, razi hospital, manouba, Tunisia

## Abstract

**Introduction:**

Treatment-resistant schizophrenia remains a major challenge in psychiatry due to its resistance to conventional therapeutic interventions, leading to persistent symptoms and functional impairment. Efforts to address it focus on exploring alternative approaches, including novel medications, psychosocial interventions, and neurostimulation techniques.

**Objectives:**

To determine the sociodemographic and clinical profile of patients with treatment-resistant schizophrenia.

**Methods:**

We conducted a retrospective descriptive study involving patients diagnosed with treatment-resistant schizophrenia at Psychiatry Service D of Razi Hospital in Tunisia over a five-year period (2019-2023). Data collection was conducted retrospectively by initially referring to medical records and subsequently verifying and supplementing information through patient or family interviews. Sociodemographic variables included age, gender, educational level, socioeconomic status, marital status, and occupation. Clinical data of interest comprised age at onset of mental disorder, duration of untreated psychosis, illness duration, family history of mental illness, previous treatments and their dosages, medical and psychiatric comorbidities, therapeutic adherence, and clinical scores using the Positive and Negative Syndrome Scale (PANSS).

**Results:**

The sample size is 30 patients.

We identified a distinct demographic and clinical profile among patients diagnosed with treatment-resistant schizophrenia. The cohort exhibited a notable male predominance, constituting 60% (n=18) of the study population, with a mean age of 39 ± 14.58 years. The majority of patients were unmarried (70%, n=21) and belonged to a lower socioeconomic status (80%, n=24).

In terms of clinical characteristics, patients experienced an early onset of the disorder at 18 ± 3.58 years, coupled with a prolonged duration of untreated psychosis averaging 8 ± 2.65 years. There was a substantial prevalence of family history of mental illness among the cohort (80%, n=24).

The initial psychiatric assessment, based on PANSS scores obtained from the first hospitalization, revealed elevated symptomatology levels: Positive subscale score averaged 28 ± 5.23, Negative subscale score averaged 20 ± 4.53, and General psychopathology score averaged 65 ± 10.02.

**Conclusions:**

Comprehensive clinical and sociodemographic profiling of individuals with treatment-resistant schizophrenia is essential for enhancing clinical outcomes and tailoring treatment strategies. This approach allows clinicians to better understand the unique challenges these patients face, enabling the development of more targeted and effective interventions to improve their quality of life.

**Disclosure of Interest:**

None Declared

